# HIV-2-Infected Macrophages Produce and Accumulate Poorly Infectious Viral Particles

**DOI:** 10.3389/fmicb.2020.01603

**Published:** 2020-07-10

**Authors:** Ester Gea-Mallorquí, Laurent Zablocki-Thomas, Mathieu Maurin, Mabel Jouve, Vasco Rodrigues, Nicolas Ruffin, Philippe Benaroch

**Affiliations:** ^1^Institut Curie, PSL^∗^ Research University, INSERM U932, Paris, France; ^2^Institut Curie, PSL^∗^ Research University, UMR3216, Paris, France

**Keywords:** macrophages, HIV-2, viral assembly, virus-containing compartment, restriction factors, HIV transmission to T cells, viral reservoir

## Abstract

A significant proportion of HIV-2-infected patients exhibit natural virological control that is generally absent from HIV-1-infected patients. Along with CD4^+^ T cells, HIV-1 targets macrophages which may contribute to viral spreading and the latent reservoir. We have studied the relationship between macrophages and HIV-2, focusing on post-entry steps. HIV-2-infected monocyte-derived macrophages (MDMs) produced substantial amounts of viral particles that were largely harbored intracellularly. New viruses assembled at the limiting membrane of internal compartments similar to virus-containing compartments (VCCs) described for HIV-1. VCCs from MDMs infected with either virus shared protein composition and morphology. Strikingly, HIV-2 Gag was mostly absent from the cytosol and almost exclusively localized to the VCCs, whereas HIV-1 Gag was distributed in both locations. Ultrastructural analyses of HIV-2-infected MDMs revealed the presence of numerous VCCs containing both immature and mature particles in the lumen. HIV-2 particles produced *de novo* by MDMs were poorly infectious in reporter cells and in transmission to activated T cells through a process that appeared independent of BST2 restriction. Rather than being involved in viral spreading, HIV-2-infected macrophages may represent a cell-associated source of viral antigens that can participate in the immune control of HIV-2 infection.

## Introduction

Macrophages are key players of the innate immune system and can rapidly respond to many pathogens. In HIV-1 infection, macrophages are also targets in which the virus replicates and accumulates. Infected macrophages tend to resist the cytopathic effects of HIV-1, can survive for long periods of time (Igarashi et al., 2001; Swingler et al., 2007) and have been found in many tissues from infected individuals (Cribbs et al., 2015). The role of macrophages in the physiopathology of the HIV infection has long been debated (Wacleche et al., 2018; Kruize and Kootstra, 2019), but they are known to be involved in the brain, with the development of HIV-1-associated neurological disorders (Koppensteiner et al., 2012).

The importance of macrophages in the formation of a viral reservoir in infected patients has received support from several studies performed in macaques and humanized mice (Honeycutt et al., 2016, 2017; Araínga et al., 2017). HIV-1 infected tissue macrophages have been recently found in the liver and urethra in samples from individuals undergoing cART (Kandathil et al., 2018; Ganor et al., 2019). The persistent inflammation observed in HIV-1-infected patients also appears to involve macrophages (Clayton et al., 2017). A large body of work has characterized the HIV-1 life-cycle in macrophages (see Rodrigues et al., 2017), the impact of the infection on macrophage function (Mazzolini et al., 2010; Verollet et al., 2014), and the sensing mechanisms involved (Chattergoon et al., 2014; Decalf et al., 2017; Nasr et al., 2017). In contrast to HIV-1, very few studies have evaluated the role of macrophages in HIV-2 infection.

HIV-2 is the second causative agent for AIDS and leads to an immunodeficiency syndrome that is essentially identical to that of HIV-1 once CD4^+^ T cell counts drop. Both viruses share comparable genetic organization, structural architecture, and most virological features. Despite this close relationship, the clinical progression of HIV-2 to AIDS is much slower compared to HIV-1 and may reflect better immune control of the infection (Marlink et al., 1994; Nyamweya et al., 2013; Esbjörnsson et al., 2019). A much larger proportion of people with HIV-2 infection exhibit long-term viral control without ART than is seen in HIV-1 infection (van der Loeff et al., 2010).

It is not yet understood how the better control of HIV-2 infection is induced and maintained. Specific features of the HIV-2 replication cycle may contribute to the differences observed in the pathophysiology. The proviral loads (the number of copies of HIV DNA integrated into circulating CD4^+^ T cells) in infected patients are similar for the two viruses (Rowland-Jones and Whittle, 2007). In contrast to HIV-1, the number of free circulating viral particles estimated as the circulating viral load in plasma is substantially lower and usually undetectable in HIV-2-infected individuals (Popper et al., 2000; Damond et al., 2002).

Lower levels of circulating virus have been associated with a decreased rate of vertical and horizontal transmission in HIV-2 compared to HIV-1 (Nyamweya et al., 2013). Although, it is estimated that 30–40% of HIV-2 infected people have low or undetectable viral loads (van der Loeff et al., 2010), ongoing viral replication still occurs in HIV-2-infected patients (Soares et al., 2011; Fernandes et al., 2014). However, a recent longitudinal study indicates that without ART treatment, HIV-2-infected individuals have still a high probability of developing and dying from AIDS despite the longer time to progress (Esbjörnsson et al., 2019).

How the specific host immune response contributes to the low viral loads observed in HIV-2 infection remains unclear. Studying a cohort of HIV-2 long-term non-progressors from Gambia, viral control clearly correlated with the presence of a gag-specific CD8^+^ T-cell response (Leligdowicz et al., 2007). In another cohort a strong polyfunctional CD8^+^ T cell response specific for Gag was associated with HIV-2 viral control (de Silva et al., 2013). However, the Gag-specific CD8^+^ T cell responses were absent in 58% of HIV-2 viraemic individuals and the Gag-specific CD8^+^T cells exhibited features of low effector cytotoxic capacity (de Silva et al., 2013). The presence of broad and potent neutralizing antibodies in HIV-2-infected individuals at either extreme of the viral load/clinical spectrum does not correlate with progression (de Silva et al., 2012). Whether innate immune responses also contribute to HIV-2 viral control also remains to be explored.

One of the important specific features of HIV-2 particles is that they contain Vpx, an accessory protein that is absent in HIV-1. Vpx efficiently counteracts the restriction factor SAMHD1 by promoting its rapid degradation (Hrecka et al., 2011). SAMHD1 is a cytosolic enzyme active in resting T cells and DCs which depletes the stock of cytosolic dNTPs via its deoxynucleoside triphosphate triphosphohydrolase activity, thereby blocking the RT of lentiviruses (Hrecka et al., 2011; Laguette et al., 2011; Sunseri et al., 2011). The activity of SAMHD1 appears to be very low in activated CD4^+^ T cells (Descours et al., 2012), intermediate in macrophages, and high in DCs (Cribier et al., 2013). Addition of Vpx in macrophages accelerates retro-transcription and nuclear integration (Bejarano et al., 2018).

Macrophage susceptibility to HIV-2 appears to be limited by low levels of surface CD4 expression that impact Env-mediated viral fusion (Chauveau et al., 2017). Primary isolates of HIV-1 and HIV-2 appear to exhibit different replication kinetics in MDMs as judged by RT activity (Marchant et al., 2006). HIV-2 viral particles are released from MDMs, with an initial burst at day 2 post-infection (p.i.), which decreases thereafter. In contrast, HIV-1 is produced in MDMs at a low but constant rate over 21 days. HIV-2 infectivity was much lower than HIV-1 when the virus was produced in MDMs whereas infectivity was similar, yet low, when the virus was produced in PBMCs (Marchant et al., 2006). Replication kinetics and viral production in MDMs, have been estimated using HIV particles carrying their own Env together with VSV-G to bypass potential restriction at the entry step, and found for HIV-2 GL-AN to be more rapid and superior to that of the HIV-1 strains tested (Hollenbaugh et al., 2016).

There is little knowledge concerning the HIV-2 cycle in macrophages. Aside from the entry step, which relies on the highly variable Env glycoprotein, differences between the rest of the HIV-1 and HIV-2 genomes may lead to differences in the viral cycle in primary macrophages. Here we used HIV-1 and -2 viruses pseudotyped with VSV-G to normalize the entry step. We show that HIV-2-infected macrophages efficiently produced new viral particles in apparently intracellular compartments. These new viral particles were poorly transmitted to T cells, likely due to the poor quality of the viral particles produced by HIV-2-infected MDMs and the low susceptibility of CD4^+^ T cells to HIV-2. HIV-2-infected MDMs may only weakly contribute to the spread of the infection. They may, however, provide a source of cell-associated viral antigens that could stimulate the adaptive anti-viral immune response.

## Materials and Methods

### Cells

Peripheral blood mononuclear cells (PBMC) were separated from plasmapheresis residues using Ficoll-Paque (GE Healthcare). Monocytes were isolated by CD14^+^ positive selection using magnetic microbeads (Miltenyi) and differentiated into macrophages for 7 days in RPMI (Gibco, Life Technologies) supplemented with 5% fetal calf serum (FCS; BioWest), 5% human serum AB (Sigma), penicillin- streptomycin (Gibco), and 25 ng/ml macrophage colony-stimulating factor (M-CSF; ImmunoTools). CD4^+^ T cells were isolated from PBMC by negative selection using magnetic microbeads (Miltenyi).

### Cell Culture

CD4^+^ T cells were cultured in RPMI 1640 medium, GlutaMAX complemented with FBS 10% and Penicillin/Streptomycin at 10^6^ cells/mL in the presence of 5 μg/mL PHA (Lectin from *Phaseolus vulgaris* Leucoagglutinin; Sigma L2769) and 50 U/mL of IL-2 (eBioscience). On day 2 of culture, cells were washed and additionally cultured with IL-2. HEK293FT cells were cultured in DMEM medium, GlutaMAX (Thermo Fisher 61965-026) complemented with FBS 10% (Thermo Fisher 10270-106) and Penicillin/Streptomycin. GHOST X4R5 and TZM-bl cells were cultured in DMEM medium, GlutaMAX complemented with FBS 10% and Penicillin/Streptomycin. MT4C5 cells were cultured in RPMI medium, GlutaMAX complemented with FBS 10% and Penicillin/Streptomycin. All cells were cultured at 37°C with 5% CO_2_ atmosphere.

### Plasmids

HIV-1 NL4-3 (kindly provided by O. Schwartz, Institut Pasteur, Paris, France), HIV-1 NL-AD8 (Freed and Martin, 1995) derived from of NL4-3 with BaL Env, HIV-2 ROD9 and HIV-2 JK (both were gifts from N. Manel). The GFP viruses all harbor GFP instead of Nef: HIV-1 NL4-3 X4GFP (N. Manel), HIV-2 ROD9GFP and HIV-2 ROD9ΔEnvGFP (both kindly provided by O. T. Keppler), HIV-2 JKGFP (Silvin et al., 2017). HIV-1iGFP is internally tagged with EGFP sequence between MA and CA (Hubner et al., 2007). Similarly, HIV-2 ROD9GagiGFP was generated by inserting the EGFP sequence between MA and CA of Gag (gift of N. Manel). Vpu-Lai plasmid (kindly provided by S. Saragosti, Saint Louis Hospital) was used to generate Vpu-Cherry that was cloned into a pDONOR vector and transferred by gateway technology to pCDH-CMV-MCS-EF1-Puro (SBI bioscience). For lentiviral transduction, we also used pSIV3+ plasmid for Vpx, psPAX2 for lentiviral encapsidation and pDM2G for VSV-G pseudotyping. HIV-2 ROD9iGFP and HIV-2 Δψ (pSVRΔNBDM plasmid) (Lahaye et al., 2013) were co-transfected into HEK293FT cells to produce infectious particles.

### HIV-1 and Lentiviral Production and Titration

HIV viral particles were produced by transfection of HEK293FT cells in 6-well plates with 3 μg DNA and 8 μl TransIT-293 (Mirus Bio) per well. For non-pseudotyped virus 3 μg of HIV plasmid were used. For VSV-G pseudotyped virus 0.4 μg pDM2G and 2.6 μg HIV plasmid were used. For Vpx and Vpu-Cherry lentivectors 0.4 μg pDM2G, 1 μg psPAX and 1.6 μg Vpx or Vpu-Cherry plasmid were used. For production of HIV-2^∗^GagiGFP, we used a mix of 3 plasmids: 0.4 μg pDM2G, 1 μg pSVRΔNBDM and 1.6 μg pROD9GagiGFP. 16 h after transfection, media was removed, and fresh RPMI medium was added. Viral supernatants were harvested 36 h later, filtered at 0.45 μM, used freshly or aliquoted and frozen at −80°C. Viral titers were determined on GHOST X4R5 cells as described (Manel et al., 2010).

### Drugs

Azidothymidine (AZT; Sigma) was used at 25 μM final concentration and added at time of co-culture with T cells as control or after 24 h of co-culture to avoid T-to-T cell transmission.

### Flow Cytometry

Macrophages were harvested after TrypLE^TM^ Express treatment (Gibco) and gentle scraping. Cells were fixed for 20 min in 4% paraformaldehyde (PFA), washed in PBS, and when stained, permeabilized for 30 min in PBS plus 0.2% BSA and 0.05% saponin. Infection was detected by GFP coding viruses and BST2 was detected using a coupled BST2-PE antibody (eBioscience) or IgG1-PE (eBioscience) as a control and analyzed on a FACS Verse (BD). For Vpu-Cherry expressing cells CYTOFLEX cytometer (Beckman Coulter) was used.

### Recovery of Intracellular Virus

Macrophages in a 24-well plate were washed twice in PBS after supernatant collection and 400 μL of media were added and stored at −80°C. After 2 h the plate was thawed and medium was collected and centrifuged at 15,000 rpm 1 h 30 at 4°C. The pellet was resuspended in 200 μL. 100 μL were used for RNA extraction and 100 μL were diluted with 300 μL of PBS for titration into TZM-bl.

### RNA Isolation, Reverse Transcription, and qPCR

At the time of collection, supernatant was collected directly and 400 μl of supernatant and 100 μL of recovered intracellular virus were lysed with 1,400 and 350 μL of lysis buffer, respectively (Macherey-Nagel). 200 pg of Luciferase control RNA (Promega) were added to each sample for absolute quantification. RNA was collected and purified using the NucleoSpin RNA kit (Macherey-Nagel) by following the manufacturer’s instructions. RNA quality and quantity were verified with a NanoDrop 2000 (Thermo Scientific), and RT was performed using a high-capacity cDNA reverse transcription kit (Applied Biosystems) according to the manufacturer’s instructions. qPCR was performed using SYBR green Master Mix (Roche). Primers for full length gag HIV-1 and HIV-2 were designed to be in the same part of the gag sequence with similar length, Tm and % GC (HIV-2 Forward 5′-CGGCGGAAAGAAAAAGTACA-3′ and Reverse 5′-CACCAAATGACGCAGACAGT-3′; and HIV-1 Forward 5′-CGAGAGCGTCGGTATTAAGC-3′ and Reverse 5′-CTGAAGGGATGGTTGTAGCTG-3′) based on (Houzet et al., 2007) for gRNA quantification.

### Viral Genomic RNA Quantification

Standard curves of each plasmid and Luciferase RNA control (Promega) were run for each donor and plate of qPCR. The equations from the standard curves were used to calculate absolute values for HIV gRNA copies/μL with sample normalization for RNA luciferase.

### Rate of Infection on TZM-bl and Infectivity

Supernatant and recovered intracellular virus from MDMs were titrated on TZM-bl cells for 48 h using Steady-Glo Luciferase assay system (Promega) according to manufacturer’s instructions. Luminescence was read on a spectrometer (FLUOstar OPTIMA). The rate of infection (AU/μL) was calculated using the slope of the linear regression of the titration. Infectivity was calculated normalizing the rate of infection by the HIV RNA copies/μL.

### Confocal Microscopy and Immunostaining

Cells were fixed for 20 min in 4% paraformaldehyde (PFA), washed in PBS, and when stained, permeabilized for 30 min in PBS plus 0.2% BSA and 0.05% saponin. Gag was stained with H183-H12-5C hybridoma (mouse IgG1, NIH) or p24 (goat polyclonal, Abcam) only when co-stained with CD36. For cellular proteins CD44 (rat, Abcam), CD9 (rabbit, Santa Cruz), CD36 (mouse IgG1, StemCell), CD81 (mouse IgG2a, Abcam) and Lamp1 (rabbit, Abcam) were used. For secondary antibodies goat anti-IgG1 A546, goat anti-IgG2a A647, donkey anti-rabbit A488, donkey anti-goat A488, chicken anti-mouse A647, donkey anti-rabbit Cy3, donkey anti-mouse A488 and donkey anti-rat A647 were used (Life technologies).

For the Gag co-staining with different markers by immunofluorescence, single plane images (pixel size 70 nm) were acquired using an inverted confocal microscope (Zeiss, LSM780) equipped with a 40× oil immersion objective (NA = 1.3), an argon laser and three laser diodes (405, 546, and 633 nm) when required.

For the 3D % of Gag signaling measurement, Z stacks (pixel size 70 nm, Z step = 0.3 μm) were acquired using an inverted confocal microscope (Leica DMi8, SP8 scanning head unit) equipped with a 63× oil immersion objective (NA = 1.4) and four laser diodes (405, 488, 546, and 633 nm).

### Image Analysis

Image processing and analysis were performed using Fiji software (Schindelin et al., 2012). Analysis of confocal images was performed by different approaches. Line scans: the same line scan profile was applied to the different channels, profiles were performed using line profile function and normalized to their highest value before plotting the data on overlay graphs.

For the analysis of the enrichment of the different markers within VCC and cytoplasm, VCCs were segmented by applying a threshold on the Gag channel. Cytoplasm was segmented by applying a threshold on the CD44, CD36, or CD81 channel, corrected manually and subtracting the nucleus obtained after applying a threshold on the DAPI channel. The ratio between the MFI inside the VCC versus the cytoplasm for each channel was computed.

Pearson coefficients between the CD44 channel image and the Gag channel image were computed using coloc2 plugin inside the cytosol and VCC. The same process was applied for the analysis of Gag/Lamp1 co-distribution.

For the 3D volumes measurement, 3D masks were obtained for the cytoplasm, the nucleus, and the compartments by applying threshold, respectively, on CD44 channel, DAPI channel and p24 channel. Masks were corrected or adjusted manually when necessary. The volume of each masks and their GFP intensities were then measured. 3D reconstructions were achieved using Volume Viewer plugin and the 3D masks of the Fiji software.

### Electron Microscopy

Cells were fixed in 2% glutaraldehyde in 0.1 M cacoldylate buffer, pH 7.4 for 1 h, post-fixed for 1 h with 2% buffered osmium tetroxide, dehydrated in a graded series of ethanol solution, and then embedded in epoxy resin. Images were acquired with a digital camera Quemesa (SIS) mounted on a Tecnai Spirit transmission electron microscope (FEI Company) operated at 80 kV. Density of viral particles was determined as viral particles/μm^2^ within VCC. Measurement of VCC area was done using iTEM analySIS software (Soft Imaging Systems).

### Macrophage-to-T Cell Transmission/MT4C5

For Macrophage-to-T cell transmission, MDMs in 96-well plate and 24-well plate were infected with VSV-G–pseudotyped HIV-1 NL4.3GFP, HIV-2 ROD9GFP, and HIV-2 JKGFP at different MOIs, washed extensively 3 days later, and received 7.5 × 10^5^ CD4^+^ T lymphocytes/well in the 96-well plate. MDMs on 24-well plate were harvested for the measurement of infected cells by flow cytometry. The CD4^+^ T lymphocytes were purified by negative selection (Miltenyi Biotec) from heterologous PBMCs that had been activated 48 h before in RPMI, 10% FCS, 2.5 μg/ml PHA-L, and 30 U/ml IL2. 25 μM AZT was added at 0 h (as a negative control) or 24 h after the beginning of the co-cultures. At 48 h, CD4^+^ T cells were collected, washed and fixed with 4% PFA, permeabilized, stained with PE-Cy7 anti-CD3 (BD), and analyzed on a FACS Verse (BD).

For Macrophage-to-MT4C5 cell transmission, MDMs were infected 96-well plate and 24-well plate with VSV-G–pseudotyped HIV-1 NL4.3GFP, HIV-2 ROD9GFP, HIV-2 JKGFP, and HIV-2ΔEnv ROD9GFP at MOI 1.5, washed extensively 8 h later and at day 3, when received 7.5 × 10^5^ MT4C5 cells/well in the 96-well plate. MDMs on 24-well plate were harvested for % of infected cells, and supernatant was harvested for gRNA and infectivity quantification. 25 μM AZT was added at 24 h after the beginning of the co-cultures. At 48 h, MT4C5 cells were collected, washed and fixed with 4% PFA and analyzed on a FACS Verse (BD). Data were analyzed using FlowJo v10 and Prism v8 for Mac (GraphPad).

### Statistics

Data were analyzed using Prism v8 for Mac (GraphPad). Tests for statistical significance were chosen accordingly to the data and assuming non-parametric distribution. Applied tests are specified for each figure in the legend.

## Results

### HIV-2 Infection and Production in Primary Macrophages

**FIGURE 1:**
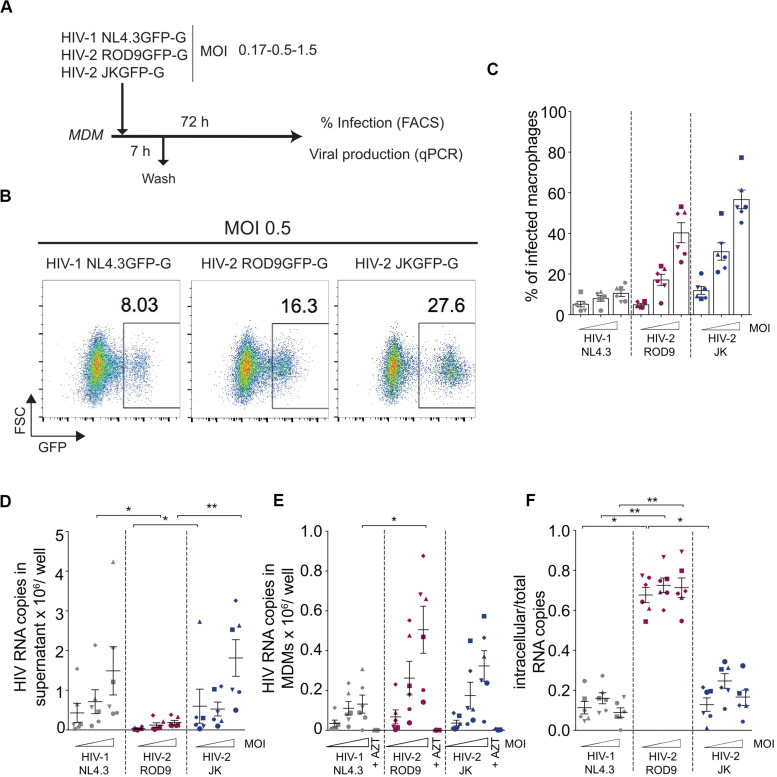
HIV-2 infection and production of MDMs. **(A)** Outline of the experiment. Monocyte-derived macrophages were differentiated for 7 days with M-CSF (MDMs), infected with the indicated viruses for 7 h at different MOIs and harvested at 3 dpi for analysis of GFP expression by flow cytometry and viral genomic RNA quantification. **(B)** Representative dot plot analyses of infected MDMs with percentages of GFP^+^ cells indicated in the plots. **(C)** Percentages of GFP^+^ MDMs infected with the indicated viruses at 3 dpi. **(D)** Viral release in the supernatant and **(E)** Intracellular viral content at 3 dpi of MDMs evaluated by RT-qPCR. **(F)** Ratio of intracellular viral copies over total viral copies produced by infected MDMs. *n* = 6 from 3 independent experiments, individual data (each symbol representing one MDM donor across the panels) displayed with means ± standard deviations (SD). *P*-values were calculated using non-parametric Friedman paired test. *P*-values lower than 0.05 were considered as significant (*p < 0.05, **p < 0.01, ***p < 0.001, ****p < 0.0001).

We used HIV-1 and -2 viruses encoding GFP in place of Nef to quantify the rate of infection in MDMs, independently of antibodies which may have differential affinity for HIV-1 and -2 proteins. The rate of infection determined by assays based on the RT activity of both viruses was not considered because of the viruses’ differential catalytic activity and sensitivity to inhibitors (De Clercq, 1993; Achuthan et al., 2017). VSV-G pseudotyped HIV-1 NL4.3GFP, HIV-2 ROD9GFP, and HIV-2 JKGFP were produced in HEK293FT cells by transfection and titrated on GHOST reporter cells for use at similar MOIs on MDMs (Figure 1A). Infection rates were consistently lower for HIV-1 than HIV-2 (Figures 1B,C). This likely resulted from the presence of Vpx in HIV-2 particles, which counteracts SAMHD1 activity. As previously shown (Hrecka et al., 2011; Sunseri et al., 2011), the rate of MDM infection by HIV-1 increased roughly 10-fold when the virus was complemented with Vpx (Supplementary Figure S1), showing that our preparations of HIV-1 were functional. Direct comparison of Gag amounts by flow cytometry or immunoblot was not reliable due to differences in antibody affinity for HIV-1 and -2 proteins. To accurately follow viral production by MDMs infected with the different viruses at three MOIs, we quantified HIV RNA copies at both the intra- and extracellular levels after 3 days of infection (Figures 1D,E). HIV-1-infected MDMs released increasing amounts of viral RNA copies in the supernatant as a function of the MOI (Figure 1D). HIV-2 JKGFP-G-infected MDMs released similar amounts of viral RNA despite their higher infection rates. Levels of extracellular viral RNA were very low in ROD9GFP-G-infected MDMs (Figure 1D). However, at the intracellular level, HIV-2 ROD9GFP-G- and HIV-2 JKGFP-G-infected MDMs contained the highest amounts of viral RNA (Figure 1E).

To estimate the level of cell-associated virus, we calculated the ratio of intracellular to total viral RNA copies (Figure 1F). HIV-1 NL4.3GFP and HIV-2 JKGFP exhibited similar low ratios whereas the ratio was higher for HIV-2 ROD9GFP, reflecting its low release from MDMs. We concluded that both HIV-2 strains contained high intracellular concentrations of viral RNA despite their differences in viral release.

### HIV-2 Is Produced and Stored in HIV-1-Like Virus-Containing Compartments

**FIGURE 2:**
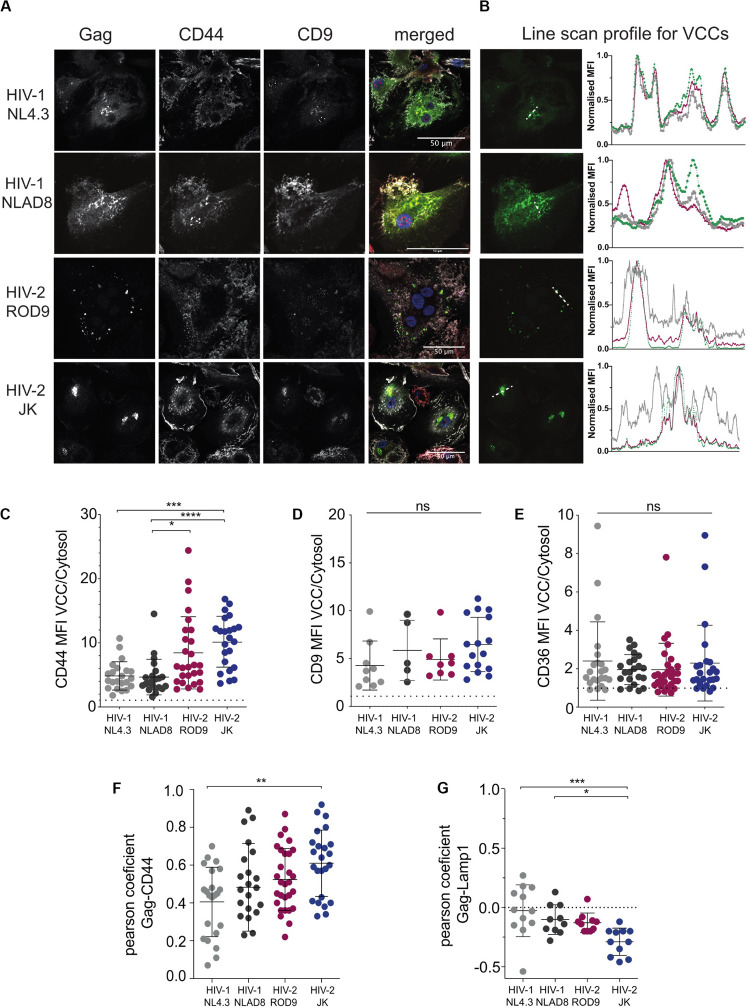
HIV-2 Gag localizes in Virus-Containing Compartments in primary human MDMs. **(A)** MDMs were infected for 3 days with the indicated viruses, all VSV-G-pseudotyped. Samples were stained for the indicated markers by immunofluorescence. Representative confocal sections are presented from at least 6 donors. **(B)** Line intensities profiles for all the markers **(A)**. Profiles were performed on the white dotted lines shown on the Gag images. Intensities profiles were normalized to highest value for each marker. Green line corresponds to Gag, gray to CD44 and red to CD9. **(C–E)** Quantification of each marker enrichment in VCCs calculated as the ratio MFI present in VCCs over the one present in the cytosol on confocal sections. VCC and cytosol were segmented by masks on Gag and CD44 staining, respectively (see section “Materials and Methods”). **(F)** Analysis of the co-distribution between Gag and CD44 and **(G)** between Gag and Lamp1 on confocal sections by measurement of the Pearson coefficient. Each dot represents a cell. *P*-values were calculated using Kruskal–Wallis non-parametric test with Dunn’s correction for multiple comparison. *P*-values lower than 0.05 were considered as significant (*p < 0.05, **p < 0.01, ***p < 0.001, ****p < 0.0001).

To determine where viral production occurred in HIV-2-infected MDMs, we first analyzed the intracellular distribution of the viral Gag protein by immunofluorescence. Here we used VSV-G pseudotyped WT viruses and extended our analyses to R5 virus HIV-1 NLAD8, as HIV-2 JK is also R5 tropic whereas both HIV-1 NL4.3 and HIV-2 ROD9 are X4 tropic. Confocal microscopy revealed the presence of punctate structures containing high amounts of Gag in MDMs infected with HIV-1 or -2 (Figure 2A). Line scan profiles through Gag^+^ containing compartments showed the co-enrichment of CD44 and CD9 for the four viral strains in those structures (Figure 2B). CD44 staining was intense at the plasma membrane and dimmer intracellularly where it was associated with Gag mainly in the punctuate structures (Figures 2A,B). Pearson coefficient analysis of the co-distribution of both markers in the cell showed only partial co-localisation (Figure 2F). The Gag-containing structures were free of Lamp1 (see Supplementary Figure S2A and Figure 2G). We also estimated the level of enrichment of CD44, CD9, and CD36 by measuring their staining intensities in the cytosol versus in the Gag^+^ structures, and observed an enrichment in all conditions for the three markers (Figures 2C–E). We extended these quantifications to include CD81 and more donors comparing HIV-1 NLAD8 and HIV-2 ROD9 and obtained similar results (Supplementary Figures S3A–C). Similar staining distributions were also observed at day 7 p.i. (Supplementary Figure S2B). We concluded that Gag concentrated into CD44^+^CD9^+^CD36^+^CD81^+^Lamp1^–^ compartments in MDMs infected by the HIV strains used. Such composition is typical of VCCs previously described in HIV-1-infected MDMs (Pelchen-Matthews et al., 2003; Deneka et al., 2007; Jouve et al., 2007; Bèrre et al., 2013). These findings suggest that in MDMs, like HIV-1, both HIV-2 strains hijack these compartments for their assembly.

### Gag Is Almost Absent From the Cytosol but Concentrated in VCCs in HIV-2-Infected Macrophages

**FIGURE 3:**
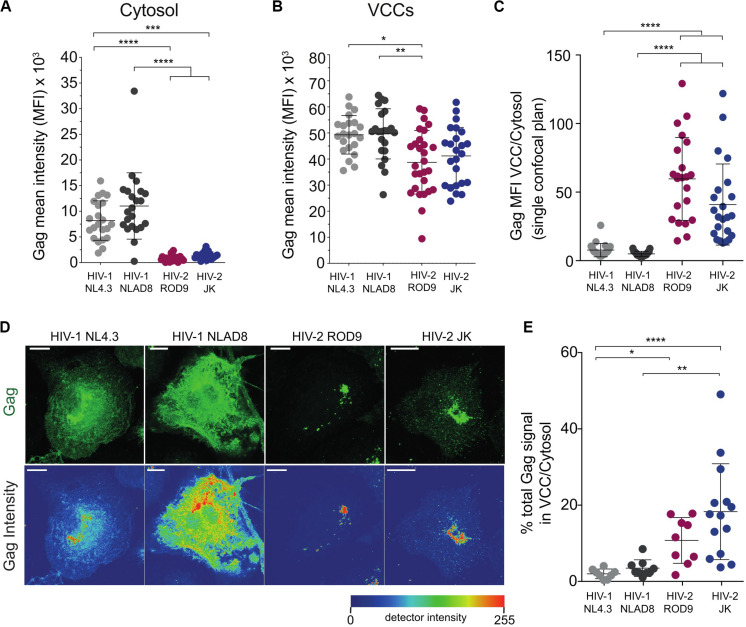
Gag is concentrated in the viral-containing compartment of HIV-2-infected MDMs. **(A,B)** Gag MFI in the cytosol **(A)** and in the VCCs **(B)**. A macro was used to build masks that delimited the VCCs based on Gag and the Cytosol based on CD44 plasma membrane staining (see section “Materials and Methods”). **(C)** Gag enrichment in the VCCs calculated as the ratio of the MFI in VCCs **(B)** over the MFI in the cytosol **(A)** on confocal sections. **(D)** Representative Z projections of a stack of images obtained by confocal microscopy. Images displayed on the second row represent intensity maps for Gag expression using pseudo-color. Bar = 10 μm. **(E)** Z stacks of infected MDMs were used to obtain 3D masks and to determine the % of Gag signal present in VCCs over Gag signal present in the rest of the cells. Each dot represents a cell. Data presented from a representative donor from at least 6. *P*-values were calculated using Kruskal–Wallis non-parametric test with Dunn’s correction for multiple comparisons. *P*-values lower than 0.05 were considered as significant (*p < 0.05, **p < 0.01, ***p < 0.001, ****p < 0.0001).

Gag staining in HIV-2-infected MDMs appeared to be mainly found in punctate structures that were likely to be VCCs (Figure 2A). In contrast, HIV-1 Gag accumulated in VCCs and was also present in the cytosol (Figure 2A). Quantification on confocal sections of Gag signal present in the cytosol, in VCC and their ratio (Figures 3A–C, respectively) confirmed this striking difference between HIV-1 and -2 Gag distribution that was observed at day 3 p.i. (Figures 2A, 3A–C) and persisted to day 7 p.i. (Supplementary Figure S2B). Quantification on larger number of MDMs from different donors confirmed these differences in Gag distribution (Supplementary Figure S4A). To better show the differences in Gag localisation, we generated z projections of confocal z-stacks of infected MDMs to reconstruct in 3D the Gag^+^ compartments, i.e., the VCC (see z projections in Figure 3D). Analysis of the Gag signal distribution, shown in pseudo-colors to visualize very low levels, confirmed that Gag was mostly absent from the cytosol in HIV-2-infected MDMs and concentrated in VCCs (Figure 3D). This conclusion was further supported by quantification of the Gag signal present in VCCs/in the whole cell, in 3D stacks of images of HIV-infected MDMs (Figure 3E).

To rule out that the differential distribution of HIV-1 and HIV-2 Gag was due to differential affinity of the H183-H12-5C mAb used for the cytosolic Gag forms, we infected MDMs with HIV strains internally tagged with GFP. The GFP was inserted between the matrix and capsid within the p55 Gag precursor between two protease sites (see Hubner et al., 2007 and methods). The GFP signal and Gag staining were co-distributed in HIV-1iGFP- and HIV-2^∗^iGFP-infected MDMs (Supplementary Figure S4B), confirming the differential distribution of Gag.

The addition of Vpx to HIV-1 NL4.3GFP-G increased the infection rate of MDMs as expected (Supplementary Figure S1A), but did not modify Gag distribution (Supplementary Figures S4C,D). These data suggest that both HIV-2 strains ROD9 and JK Gag were more efficiently recruited to VCCs after synthesis in the cytosol than HIV-1 Gag.

### Ultrastructural Analysis of VCCs From HIV-2-Infected Macrophages

**FIGURE 4:**
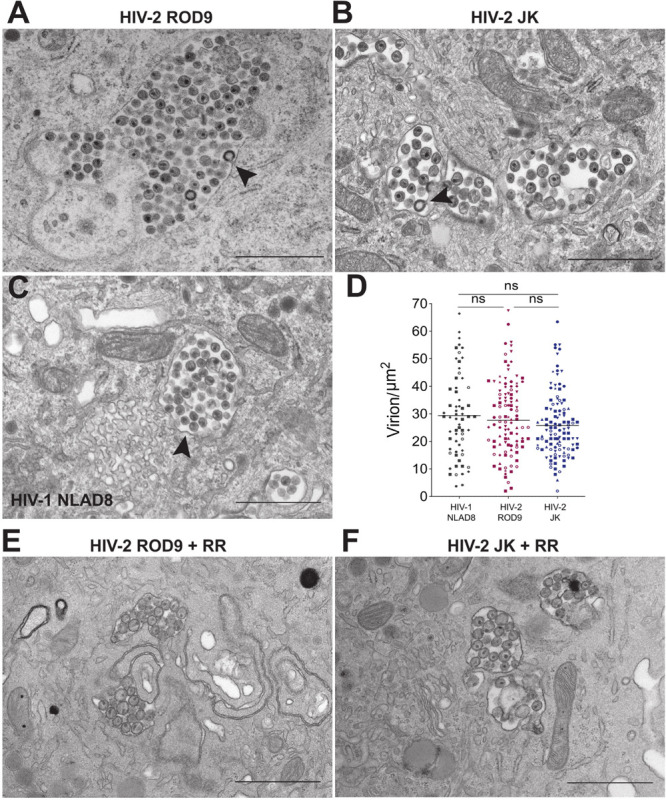
HIV-2 assembles and buds in *bona fide* VCC of MDMs. **(A–C)** MDMs infected with the indicated pseudotyped viruses for 3 days were fixed and processed to perform electron microscopy (see section “Materials and Methods”). A representative epon section of infected MDM from 5 donors is presented. Arrowheads point to nascent viral buds. **(D)** Quantification of the density of viral particles present within the VCCs calculated for each epon sections of infected MDMs. Each dot represents a VCC. Bars represent Grand means. Donors: NLAD8 *n* = 4, ROD9 *n* = 7, and JK *n* = 5. **(E,F)** MDMs infected with the indicated pseudotyped viruses for 3 days were fixed in the presence of Ruthenium Red and processed to perform electron microscopy. *P*-values were calculated using Kruskal–Wallis non-parametric test with Dunn’s correction for multiple comparison. *P*-values lower than 0.05 were considered as significant (*p < 0.05, **p < 0.01, ***p < 0.001, ****p < 0.0001).

We analyzed ultrathin sections of HIV-2-infected macrophages by electron microscopy to further characterize the morphology of their VCCs (Figure 4). VCCs from MDMs infected with HIV-1 NLAD8-G or HIV-2 ROD9-G or HIV-2 JK-G exhibited highly similar morphologies. In all cases we observed viral budding profiles at the VCC limiting membranes (see arrowheads), revealing the production of new virions. The budding process occurs away from the cytosol, at the limiting membrane of the VCC, and new virions accumulate in the VCC lumen. The VCCs contained mature and immature viral particles in their lumen. The presence of mature (with the electron dense capsid visible inside) and immature (with the electron dense Gag precursor at the periphery and an electron lucent zone at the center) particles revealed that they were able to mature. Quantification of the viral density in the lumen of the VCCs did not reveal any significant differences among the three types of infected MDMs (Figure 4D). We observed higher numbers of compartments per section in HIV-2-infected MDMs, probably reflecting the higher rate of infection than seen with HIV-1 (see number of dots per condition in Figure 4D).

One of the important characteristics of MDM VCCs is their connection to the extracellular medium, which can be witnessed at the ultrastructural level using the membrane impermeant dye ruthenium red (RR) (Deneka et al., 2007; Jouve et al., 2007). The RR dye decorates membranes with a characteristic electron dense staining. We performed an ultra-structural analysis of HIV-2-infected MDMs exposed to RR just before fixation and inclusion (Figures 4E,F). It revealed that the limiting membranes of VCCs from MDMs infected by both strains of HIV-2 were stained by RR as well as the membranes of the intralumenal viral particles.

These results show that HIV-2 assembled, budded and accumulated in VCCs similarly to those present in HIV-1-infected MDMs. The main difference was the paucity of cytosolic Gag in HIV-2- versus HIV-1-infected cells.

### HIV-2-Infected Macrophages Poorly Transmit the Infection to Activated Primary CD4^+^ T Cells

**FIGURE 5:**
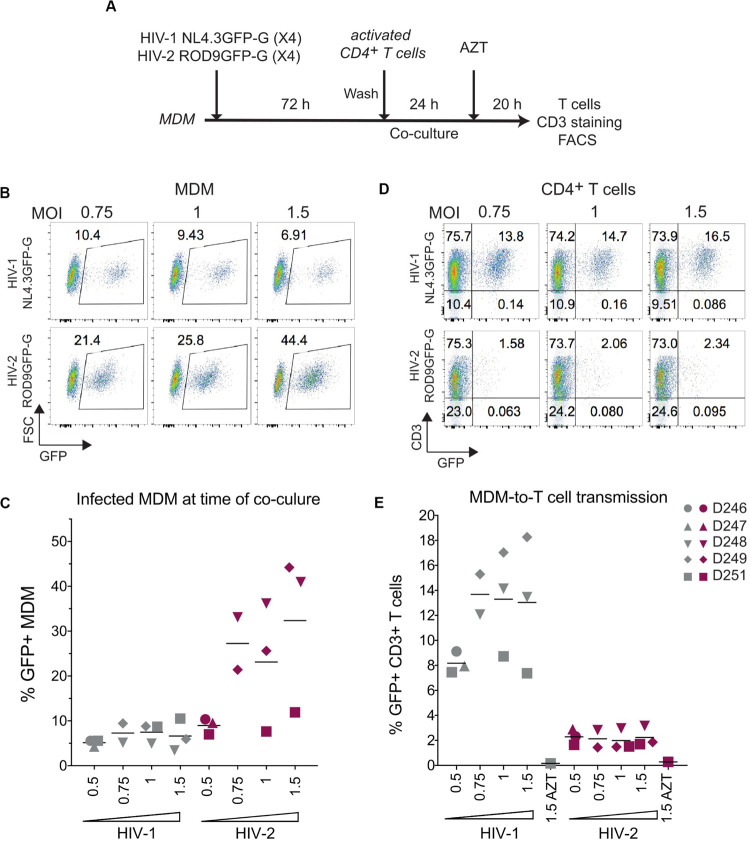
HIV-2 transmission from infected MDM to activated CD4^+^ T cells is inefficient. **(A)** Macrophage-to-T cell transmission, outline of the experiment. **(B)** Representative dot plot of GFP expression of HIV-infected MDMs analysed at 3 dpi by flow cytometry. Percentages of GFP^+^ cells are indicated in the gates. **(C)** Percentages of GFP^+^ MDMs at 3 dpi as in **(B)**, *n* = 5 donors from 3 independent experiments, bars representing the mean. **(D)** Representative dot plots of GFP and CD3 expression at the end of the 44 h co-culture. **(E)** Percentages of CD3^+^GFP^+^ T cells after co-culture with infected MDM as in **(D)**, *n* = 5 donors from 3 independent experiments, bars representing the mean.

The addition of activated T cells to HIV-1-infected MDMs has been proposed to promote polarization of VCCs and transfer of their viral contents to T cells (Gousset et al., 2008). To evaluate whether activated T cells could stimulate HIV-2-infected MDMs to release and transfer their intra-VCC viral stocks, we performed MDM-to-T cell transmission assays using HIV-1 NL4.3GFP-G and HIV-2 ROD9GFP-G, both are X4-tropic HIVs (Figure 5A). Infection of MDM at various MOIs yielded similar or higher rates of infection for HIV-2 ROD9GFP-G than HIV-1 NL4.3GFP-G (Figures 5B,C). Although HIV-1-infected MDMs efficiently transmitted the virus to T cells, transmission by HIV-2-infected MDMs was very low (Figures 5D,E).

### HIV-2 Particles Produced by MDM Are Poorly Infectious

**FIGURE 6:**
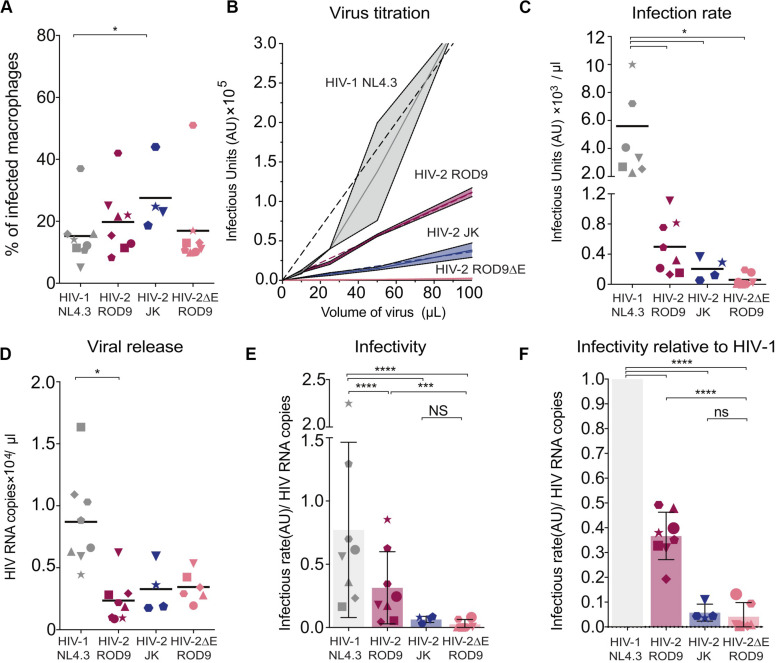
Infected MDMs produce poorly infectious HIV-2 particles. MDMs were infected with the indicated viruses. MOIs were estimated on GHOST reporter cells and adjusted to reach similar infection rates in MDMs. *n* = 8 donors from 5 independent experiments, with bars representing the mean. Each symbol represents an individual donor across all the panels. **(A)** At 3 dpi, rates of MDMs infection were determined by flow cytometry. Percentages of infected MDM, i.e., GFP^+^, at 3 dpi. **(B)** Representative virus titration on the TZM-bl reporter cell line. Serial dilutions of supernatant from each type of HIV-infected MDM were added on TZM-bl cells. Infection rates (Infectious Units/μL) were determined by calculation of the slope of the titration curve built with technical replicates. **(C)** Infection rates determined as in **(B)** with MDMs from the 8 donors. **(D)** Viral RNA copies released at 3 dpi in the supernatant of HIV-infected MDMs evaluated by RT-qPCR. **(E)** Infectivity of the released viral particles, calculated as rate of infection and normalized by genomic HIV RNA copies. **(F)** Infectivity normalized to HIV-1 for each donor. *P*-values were calculated using paired Mixed-effect analysis with Geisser–Greenhouse correction for multiple comparison test. *P*-values lower than 0.05 were considered as significant (*p < 0.05, **p < 0.01, ***p < 0.001, ****p < 0.0001).

The low transmission rates prompted us to evaluate the quality of the viruses produced by HIV-1- and HIV-2-infected MDMs at day 3 p.i. Viral production was titrated on the TZM-bl reporter cell line and normalized by quantification of gRNA. The GHOST and TZM-bl reporter cell lines allow comparable titrations, albeit TZM-bl cells are more sensitive (Supplementary Figure S5A). MDMs were infected with either HIV-1 NL4.3GFP-G, HIV-2 JKGFP-G, HIV-2 ROD9GFP-G or with HIV-2ΔEnv ROD9GFP-G as a negative control for infectivity (Figure 6A). Infection rates of both HIV-2 strains were much lower than that of HIV-1, and were null for HIV-2ΔEnv as expected (Figures 6B,C). Combined with the estimation of the viral content of the supernatant (Figure 6D), the infectious capacity of the HIV-2 viruses produced by MDMs was very low (Figures 6E,F): less than 50% of the infectivity of HIV-1 for HIV-2 ROD9GFP, and 20% for HIV-2 JKGFP.

Our data suggest that viral particles produced by HIV-2-infected MDMs possess reduced infectivity compared to HIV-1, as judged on TZM-bl reporter cells. In addition, HIV-2 WT particles produced by HEK293FT cells infected CD4^+^ T cells less well compared to HIV-1 (Supplementary Figure S5), even when compared at the same GHOST-determined MOI (see vertical boxes Supplementary Figure S5C). Low HIV-2 infection rates of T cells observed in the transmission experiments appear to result from the low infectivity of the HIV-2 particles produced by infected MDM combined with the low susceptibility of CD4^+^ T cells to HIV-2.

### BST2 Regulation in HIV-Infected Macrophages

The low amounts of HIV-2 ROD9 released and the low infectivity of HIV-2 particles produced by MDMs may result from partial down-regulation of the restriction factor BST2/Tetherin. BST2 affects viral release and infectivity of newly produced viral particles (Neil et al., 2007). It is counteracted by the small accessory HIV-1 protein Vpu by promoting its removal from the plasma membrane in T cells and further degradation (Neil et al., 2008). Vpu is absent from HIV-2 but its Env glycoprotein can sequester BST2 into the *Trans*-Golgi Network, as shown so far only in HeLa cells (Le Tortorec and Neil, 2009; Hauser et al., 2010).

**FIGURE 7:**
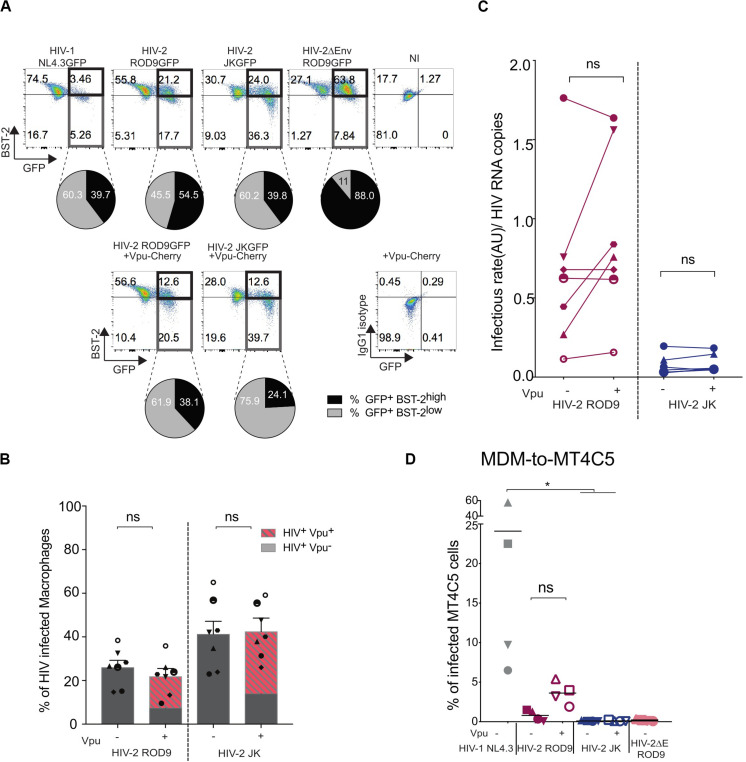
Vpu-induced downregulation of BST2 does not rescue infectivity of the viral particles produced by HIV-2-infected MDMs. MDMs were infected with the indicated HIV or not (NI = non-infected) supplemented with lentiviral particles encoding the fusion protein Vpu-Cherry. At 3 dpi, cells were recovered, fixed and stained for BST2. **(A)** Representative dot plot of BST2 versus GFP expression. GFP^+^BST2^*low*^ cells are boxed to ease comparisons. Pie charts represent BST2 expression within the infected GFP^+^ cells. Isotype control staining is shown at the bottom right. **(B)** Rates of infection were evaluated by measuring the percentage of GFP^+^ MDM exposed or not to Vpu-Cherry lentiviral particles. Percentages of GFP^+^ cells and of GFP^+^Vpu^+^ (in dashed red) are displayed. Each symbol represents a donor (*n* = 7 from 4 independent experiments). Bars represent the mean ± SEM. **(C)** Infectivity of the released viruses +/– Vpu, calculated as rate of infection/HIV RNA copies measured as Figure 5E (see also Supplementary Figure S5). For **(B,C)** *P*-values were calculated using matched-pairs Wilcoxon non-parametric test. *P*-values lower than 0.05 were considered as significant. **(D)** Addition of Vpu to HIV-2 ROD9-infected MDMs partially rescues transmission to MT4C5 cells. Percentages of GFP^+^ MT4C5 cells, which is a CD4^+^CCR5^+^CXCR4^+^ T cell line, after co-culture with MDMs infected with indicated HIV +/– lentiviral particles encoding Vpu-Cherry. *n* = 4 from 2 independent experiments with bars representing the mean. *P*-values were calculated using paired Friedman non-parametric multiple comparison test. *P*-values lower than 0.05 were considered as significant (*p < 0.05, **p < 0.01, ***p < 0.001, ****p < 0.0001).

HIV-2 ROD9 possesses an Env glycoprotein with a short cytoplasmic tail and carries point mutations at sites described to be important for BST2 counteraction (Bour and Strebel, 2003). Nevertheless, HIV-2 ROD9-infected MDMs were able to downregulate BST2 as determined by flow cytometry on fixed and permeabilized cells, although BST2 downregulation was stronger with HIV-1 and HIV-2 JK (Figure 7A). We checked that HIV-2 JK Env possesses a complete cytoplasmic tail and none of the mutations described to compromise BST2 counteraction. While both HIV-2 strains downregulated BST2 expression, HIV-2 ROD9ΔEnv did not (Figure 7A). We concluded that HIV-2 infection of MDMs was accompanied by an Env-mediated downregulation of total BST2 expression.

To better counteract BST2, we exposed MDMs to both HIV-2 and lentivirus carrying HIV-1 Vpu fused to mCherry to induce a HIV-1-like BST2 downregulation. Vpu-Cherry was expressed in 84% (for HIV-2 ROD9) and 77% (for HIV-2 JK) of infected MDMs (Figure 7B). Expression of Vpu-mCherry reduced BST2 levels, which became similar to or lower than those observed in HIV-1- infected MDMs (Figure 7A and Supplementary Figure S6A). However, Vpu-Cherry expression did not significantly impact on the infectivity of the particles produced by MDMs (Figure 7C and Supplementary Figures S6B,C), nor did it affect MDM-to-T cell transmission using a CD4^+^ T cell line expressing both HIV co-receptors (Figure 7D).

We concluded that the reduced release for ROD9 and weak infectivity of both HIV-2 strains produced by MDMs cannot be or could only marginally be ascribed to BST2-mediated restriction.

## Discussion

The involvement of macrophages in the pathophysiology of HIV-1 infection has recently been highlighted (Honeycutt et al., 2016, 2017; Araínga et al., 2017). Infected macrophages have even been found in individuals under cART in the liver and the urethra (Kandathil et al., 2018; Ganor et al., 2019). HIV-1-infected macrophages are likely to fuel the maintenance of the viral reservoir in infected patients under anti-retroviral therapy due to their resistance to the cytopathic effect of the virus, their capacity to store infectious particles and their localization in tissues. In contrast to HIV-1, knowledge concerning the relationship between macrophages and HIV-2 is scarce. We undertook the present study using an *in vitro* model of primary human MDMs and focused on the post-entry steps to evaluate whether HIV-2 possesses any intrinsic features that could contribute to its low pathogenicity and better immune control.

We focused on the late stages of the HIV-2 viral cycle in macrophages by normalizing the entry step by pseudo-typing viral particles with VSV-G. All our results were obtained with only two HIV-2 strains and therefore might not be generalized to other HIV-2 strains that are genetically diverse (Gao et al., 1994; James et al., 2019). Our data show that upon entering macrophages HIV-2 replicated better than HIV-1, probably due to the presence of Vpx in HIV-2 that efficiently counteracts SAMHD-1 restriction. HIV-2 assembled, bud and accumulated in the same apparently intracellular compartments, named VCCs, as HIV-1. The composition and the morphology of the VCCs appeared to be similar for both viruses based on the presence of specific markers and our ultrastructural analyses. Comparative proteomic analysis of the HIV-1 and HIV-2 particles produced by macrophages would be informative as the composition of the viral membrane directly reflects that of the VCC limiting membrane (see Chertova et al., 2006).

Virus-containing compartments pre-exist in uninfected MDMs (Deneka et al., 2007; Bèrre et al., 2013) and are thought to originate from specific areas of the plasma membrane which are internally sequestered (Pelchen-Matthews et al., 2012). As a result, they often stay connected to the plasma membrane via microchannels (Deneka et al., 2007) or conduits (Bennett et al., 2009), which are too narrow for the virions that they contain to access the external medium. This access is in line with the internal neutral pH observed in the lumen of the VCC (Jouve et al., 2007). These connections allow small molecules, e.g., dextrans or dyes, such as ruthenium red (Deneka et al., 2007; Jouve et al., 2007; Gaudin et al., 2013), to rapidly access the lumen. Here we have shown at the ultra-structural level that VCCs from HIV-2 ROD9- or HIV-2 JK-infected MDMs were accessible to the ruthenium red dye, exhibited viral budding profiles and contained both immature and mature virions. These VCCs exhibited features similar to VCCs from HIV-1-infected MDMs, including their general morphology and connections with the extracellular milieu.

One striking difference between HIV-1- and HIV-2-infected MDMs concerned the intracellular distribution of the Gag precursor. HIV-2 Gag was almost absent from the cytosol and concentrated solely in VCCs, whereas HIV-1 Gag was clearly present in both locations. Quantification on 2D confocal sections and 3D reconstitutions confirmed this difference. Several hypotheses can explain these observations. Saturation of docking sites for Gag at the limiting membrane of VCC only in the case of HIV-1 Gag would account for its more abundant presence in the cytosol. Gag binding to the VCC limiting membrane probably depends on several parameters including its concentration, level of oligomerization and thus its affinity for the PI(4,5)P_2_ rich VCC membranes (Ono et al., 2004). Our data may alternatively reflect that once HIV-2 Gag is synthesized in the cytosol, it is rapidly targeted to the limiting membrane of the VCC. Two regions of the Gag precursor have been described as being key for the anchoring of Gag to the PM in T cells: the highly basic region and amino acids 84–88. The basic region comprises amino acids 16–31 (Ono, 2010) and is conserved between HIV-1 NL4.3, HIV-2 ROD9, and HIV-2 JK with only a charge change mutation at position 28 from Lys to Gln between HIV-2 and HIV-1. The 84–88 region is also different between the two viruses with a major change of Cys in HIV-2 to Ala in HIV-1 at position 84. How these few amino acid changes may impact Gag trafficking and anchoring to membranes and whether this difference in localization of Gag represents an intrinsic feature of the Gag proteins or results from the action of another viral gene product represent exciting questions for future studies.

HIV-2-infected MDMs contained high amounts of intracellular virions as seen by EM and quantification of viral RNA present in cell lysates. HIV-2 ROD9-infected MDMs released low amounts of viral particles while HIV-2 JK- and HIV-1 NL4.3-infected MDMs released similarly higher amounts. Whether the viral release from HIV-1-infected MDMs is regulated and inducible is still elusive. One of the few documented stimuli is eATP which rapidly induces viral release through a P2X7R-dependent mechanism that remains in large parts elusive (Graziano et al., 2015).

It has also been proposed that activated CD4^+^ T cells can promote the transfer of virions from VCCs of HIV-1-infected MDMs to T cells via virological synapses (Gousset et al., 2008). However, addition of activated CD4^+^ T cells to HIV-2-infected MDMs resulted in lower levels of viral transfer to T cells compared to HIV-1-infected MDMs. The numerous viral particles present in the VCCs of HIV-2-infected MDMs suggested that neosynthesis of new viral progeny was not an issue. We titrated the particles produced by HIV-2-infected MDMs and found very low titres despite our use of a sensitive reporter cell line carrying a lacZ gene driven by the HIV-2-LTR. Infected MDMs appear to produce poorly infectious HIV-2 particles.

We next hypothesized that an inefficient counteraction of the restriction factor BST2 could explain both the defect in release of HIV-2 ROD9 and poor infectivity of the viral particles. The Env glycoprotein for HIV-2, and especially its cytoplasmic tail domain, is able to downregulate BST2 just as HIV-1 Vpu is (Kueck and Neil, 2012). The precise mechanisms involved in BST2 downregulation in HIV-2 infected macrophages remain elusive. To induce an HIV-1-like counteraction of BST2, MDMs were infected with either of the two HIV-2 strains alone or together with Vpu-Cherry. This led to a further reduction of BST2 total levels. However, Vpu-Cherry expression did not significantly improve the infectivity of the virions of both HIV-2 strains tested, suggesting little to no involvement of BST2 on the phenotypes observed. The moderate effects of Vpu on BST2 downregulation and on viral production in HIV-2-infected MDMs suggested that other unidentified restriction(s) or issue(s) impacted the HIV-2 cycle in MDMs. Finally, WT HIV-2 particles produced by HEK293FT cells (that do not express BST2), poorly infected primary CD4^+^ T cells and indicated that the observed low rates of HIV-2 transfer from MDMs to CD4^+^ T cells may have resulted in part from the poor natural infectivity of HIV-2 particles.

Macrophages possess the capacity to fuse and form syncytia under normal circumstances both *in vitro* and *in vivo* (Helming and Gordon, 2009; Helming et al., 2009). The resulting multigiant cells are endowed with capacities to engulf large complement-coated targets (Milde et al., 2015). HIV-1 infection through viral Env expression promotes cell-to-cell fusion resulting in syncytia formation (Clavel and Charneau, 1994; Kondo et al., 2015). We also frequently observed syncytia formation in our cultures of HIV-1 and -2-infected-MDMs. However, the contribution of macrophages syncytia to viral spreading remains to be evaluated *in vivo*. Transmission from infected T cells to macrophages has been reported to be an efficient way to promote the formation of multi-giant cells able to sustain important viral production *in vitro* (Bracq et al., 2017). It will be of interest to compare the capacity of HIV-1 and -2 in such an experimental setting.

Activated primary DCs resist HIV-1 infection but can capture and transfer the virus to CD4^+^ T cells. In contrast, DCs are inefficient at transferring HIV-2 particles to CD4^+^ T cells (Duvall et al., 2007; Kijewski et al., 2016). Therefore, both DC and macrophages appear to be inefficient at spreading HIV-2 infection to CD4^+^ T cells. The mechanisms underlying the poor infectious capacity of the virions produced by HIV-2-infected macrophages appear complex and may involve sensing, unknown restriction- or macrophage-specific factors still yet to be identified.

The present *in vitro* study shows that HIV-2-infected MDMs can produce viral particles that accumulate in VCCs and are poorly infectious, raising the question of their role under physiological conditions. If these features are conserved *in vivo*, tissue macrophages may contribute very poorly to the spread of HIV-2 infection. Future studies should determine whether infected macrophages can present HIV-2-derived antigens and stimulate specific T cells. The capacity of macrophages to directly present viral antigens on their MHC molecules could be affected by the viral protein Nef of HIV-2, although to different extend depending on the viral strain considered (Münch et al., 2005). As recently proposed, the restriction factor (TRIM5a), which efficiently promotes the degradation of HIV-2 Gag, may generate Gag epitopes suitable for efficient presentation by infected macrophages (Boswell and Rowland-Jones, 2019). Finally, macrophages could also sense incoming HIV-2 particles and provide inflammatory signals and viral antigens to dendritic cells which could in turn elicit strong cellular immune responses.

## Data Availability Statement

The raw data supporting the conclusions of this article will be made available by the authors, without undue reservation, to any qualified researcher.

## Ethics Statement

Plasmapheresis residues were obtained from healthy adult donors (Etablissement Français du Sang, Paris, France), where all donors signed informed consent allowing the use of their blood for research purposes.

## Author Contributions

EG-M and PB designed the experiments and analysed the data. EG-M performed the experiments and built the Figures. NR set up the RT-qPCR for HIV genomic RNA quantification. LZ-T built the HIV-2^∗^GagiGFP virus. MJ performed the EM analyses. MM performed the quantitative analysis of confocal microscopy images. VR provided elements to address concerns of the reviewers. EG-M, NR, and PB discussed the results and the plan of the manuscript. PB wrote the manuscript with the contribution of EG-M and NR. All authors read and approved the final manuscript.

## Conflict of Interest

The authors declare that the research was conducted in the absence of any commercial or financial relationships that could be construed as a potential conflict of interest.

## Abbreviations

AIDS: Acquired Immune Deficiency Syndrome
AZT: azidothymidine
BST2: bone marrow stromal cell antigen 2
CA: capsid
cART: combined anti-retroviral therapy
DCs: dendritic cells
eATP: extracellular ATP
gRNA: genomic RNA
HIV-2: human immunodeficiency virus 2
MA: matrix
MDDC: Monocyte-derived dendritic cells
MDMs: monocyte-derived macrophages
MFI: mean fluorescence intensity
MOI: multiplicity of infection
PBMC: peripheral blood mononuclear cells
RT: reverse transcription
SAMHD1: SAM and HD domain containing deoxynucleoside triphosphate triphosphohydrolase 1
VCCs: virus-containing compartments
VSV-G: vesicular stomatitis virus G
WT: wild type.
