# Supplementary material for: HIV-2-Infected Macrophages Produce and Accumulate Poorly Infectious Viral Particles
Source: Front Microbiol. 2020 Jul 10;11:1603. doi: 10.3389/fmicb.2020.01603 (PMC7365954; doi:10.3389/fmicb.2020.01603)
Supplement: Supplementary file 1 [file Image_1.pdf]

Supplementary Figure S1

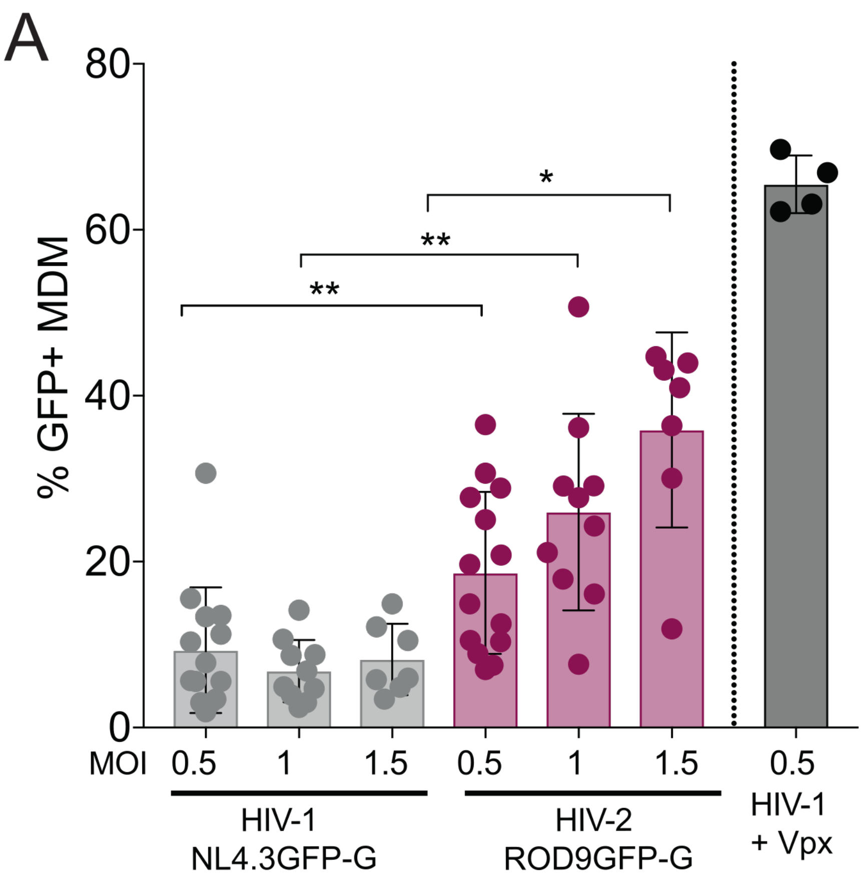

Figure S1. HIV-2 Vpx increases HIV-1 rates of infection. MDMs infected with the indicated viruses at different MOIs were harvested at 3dpi to determine their GFP expression. Vpx was delivered in separate lentiviral particles at the time of infection (see methods). P values were calculated using matched-pairs Wilcoxon non-parametric test. P values lower than 0.05 were considered as significant (*$p<0.05$; **$p<0.01$; ***$p<0.001$ and ****$p<0.0001$).
