# Supplementary material for: HIV-2-Infected Macrophages Produce and Accumulate Poorly Infectious Viral Particles
Source: Front Microbiol. 2020 Jul 10;11:1603. doi: 10.3389/fmicb.2020.01603 (PMC7365954; doi:10.3389/fmicb.2020.01603)
Supplement: Supplementary file 2 [file Image_2.pdf]

Supplementary Figure S2

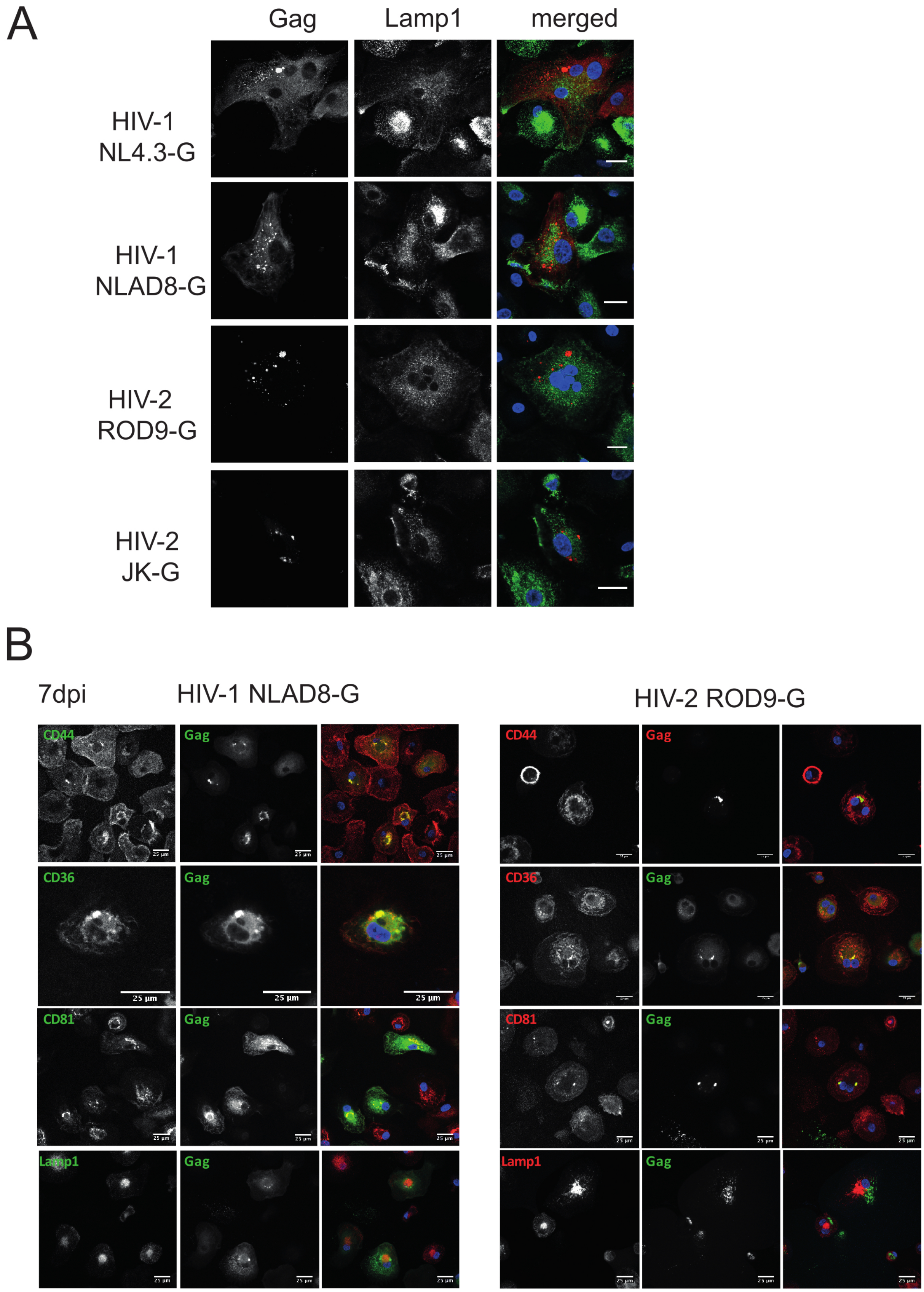

Figure S2. (A) HIV-2 Gag and Lamp1 intracellular distributions are independent. Representative confocal sections are presented for Lamp1 and Gag staining **(B) HIV-2 Gag+ compartments remain similar at day 7 post-infection.** MDMs were infected with the indicated viruses that were VSV-G pseudotyped. At 7 dpi, MDMs were stained for the indicated markers by immunofluorescence. Representative confocal sections are presented, Bar=20µm.
