# Supplementary material for: HIV-2-Infected Macrophages Produce and Accumulate Poorly Infectious Viral Particles
Source: Front Microbiol. 2020 Jul 10;11:1603. doi: 10.3389/fmicb.2020.01603 (PMC7365954; doi:10.3389/fmicb.2020.01603)
Supplement: Supplementary file 3 [file Image_3.pdf]

Supplementary Figure S3

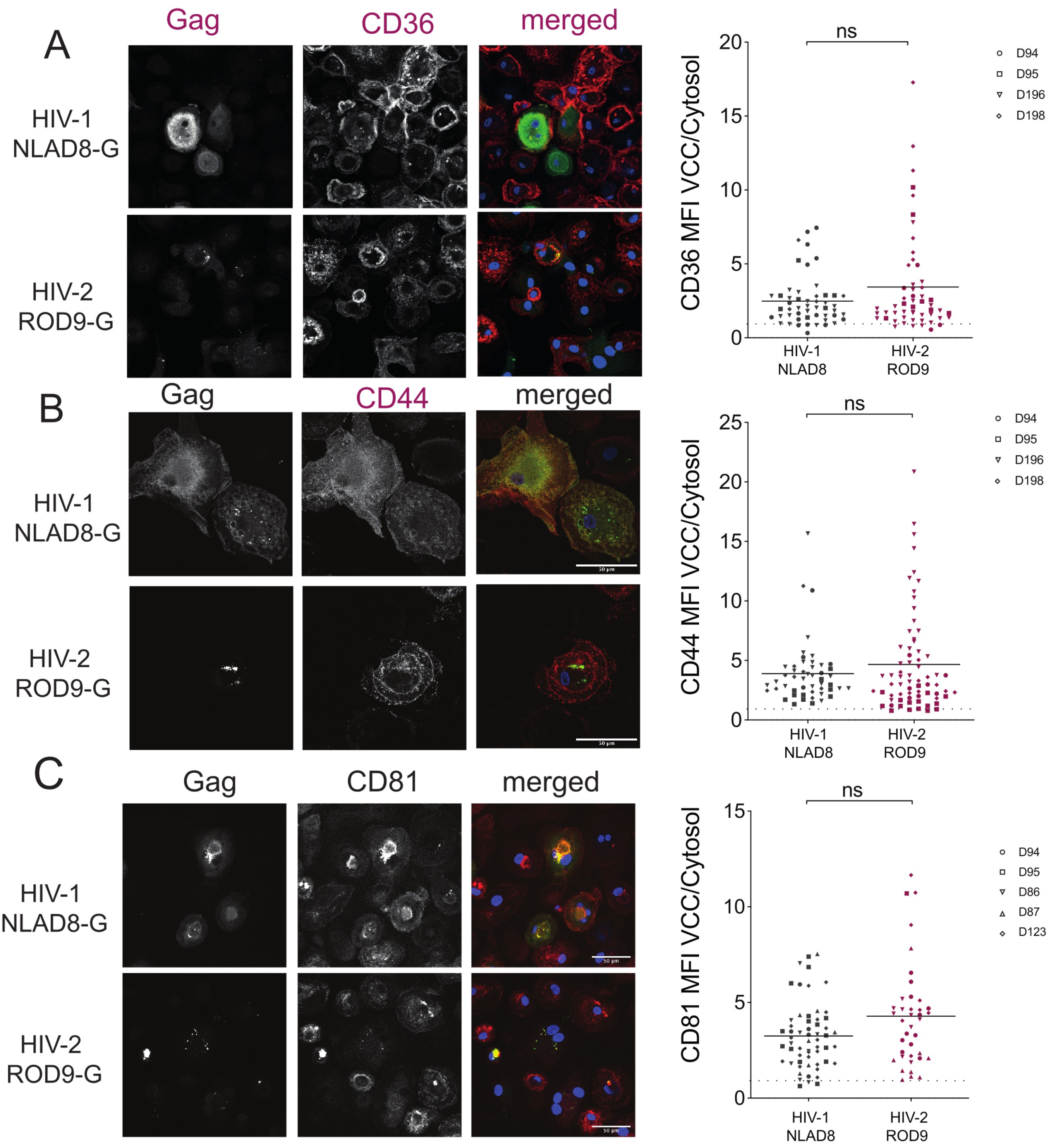

Figure S3. Comparison of Gag+ compartment in HIV-1 and -2 infected MDMs. MDMs were infected for 3 days with the indicated viruses, all VSV-G-pseudotyped. Samples were stained for the indicated markers by immunofluorescence (A, B and C). Left panels: representative confocal sections are presented. Right panels: quantification of confocal sections showing the ratio of the signal for each marker in the Gag+VCC compared to the cytosol for at least 4 different donors, each donor is represented by a different symbol. VCC and cytosol were defined by masks (see methods). P values were calculated using Mann-Whitney non-parametric test. P values lower than 0.05 were considered as significant.
