# Supplementary material for: HIV-2-Infected Macrophages Produce and Accumulate Poorly Infectious Viral Particles
Source: Front Microbiol. 2020 Jul 10;11:1603. doi: 10.3389/fmicb.2020.01603 (PMC7365954; doi:10.3389/fmicb.2020.01603)
Supplement: Supplementary file 4 [file Image_4.pdf]

Supplementary Figure S4

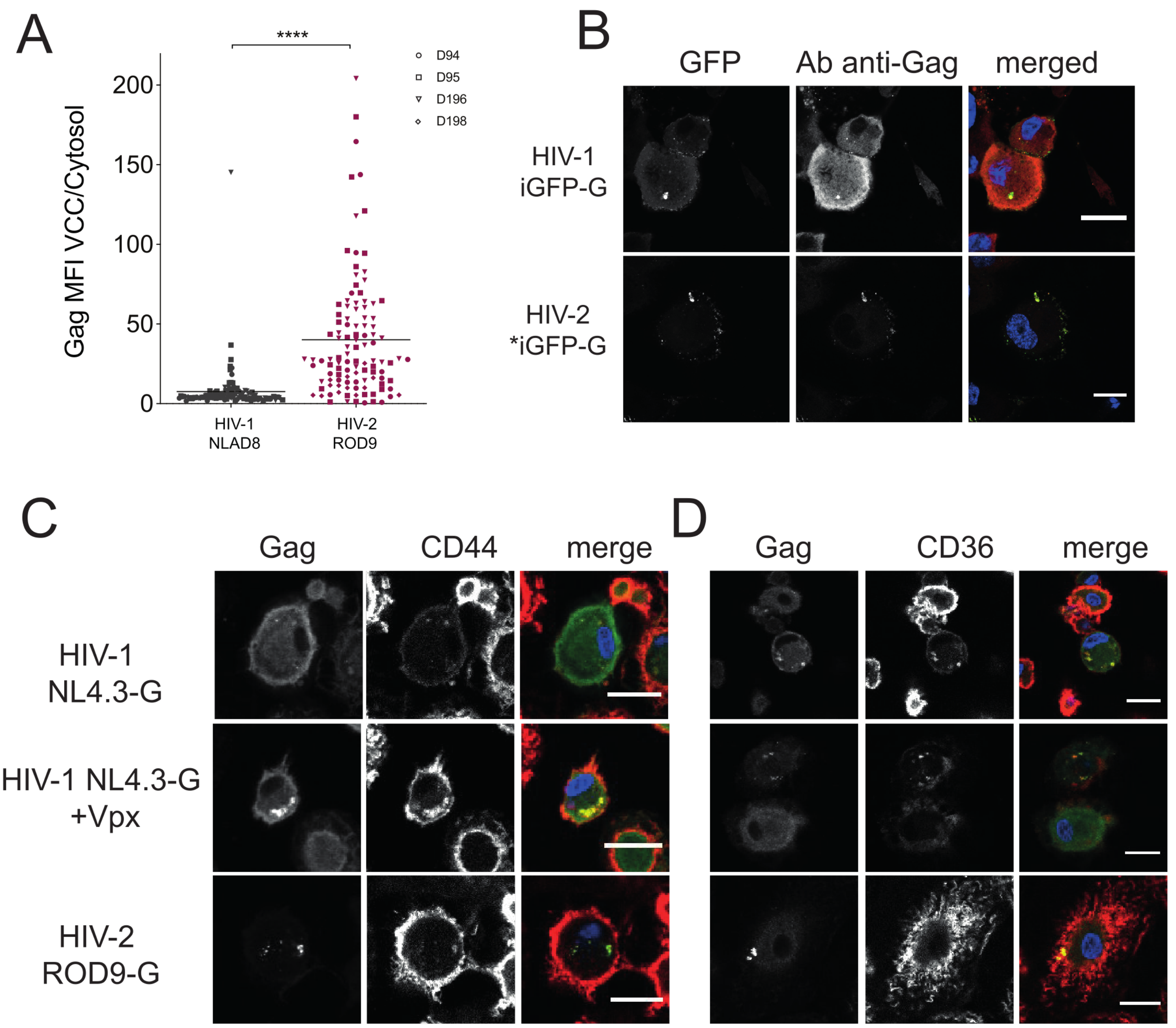

Figure S4. (A) Gag is concentrated in the Viral-Containing Compartment of HIV-2-infected MDMs. MDMs were infected for 3 days with the indicated viruses, all VSV-G-pseudotyped. Quantification for Gag concentration in the VCC was performed in cells from 4 different donors, each represented by a different symbol. Quantification of Gag concentration in VCCs calculated as the ratio MFI present in VCCs over the one present in the cytosol on confocal sections. VCC and cytosol were defined by masks (see methods). P values were calculated using Mann-Whitney non-parametric test. P values lower than 0.05 were considered as significant (*p<0.05; **p<0.01; ***p<0.001 and ****p<0.0001). **(B) GFP internally tagged HIV-2 and Gag staining co-localizes in infected MDMs.** MDMs were infected with HIV-1iGFP VSV-G or HIV-2iGFP* VSV-G complemented with HIV-2ΔPsi. Both iGFP viruses carry a GFP encoding sequence inserted between the Matrix and the Capsid of the Gag precursor that is flanked by protease sites. However, HIV-2iGFP* requires to be mixed with WT HIV-2 Gag precursor in the HEK293T cells to be properly assembled. At 3 dpi, MDMs were fixed and stained with a mAb specific for Gag. Confocal sections are presented. Bar = 20 µm **(C and D) Vpx is not responsible for HIV-2 Gag rapid localization into VCCs.** MDMs were infected with HIV-1 NL4.3 VSV-G in the presence of Vpx, or HIV-2 ROD9 VSV-G. At 3 dpi, MDMs were fixed and stained for the indicated markers. Representative confocal sections of 3 donors are presented. Bar = 20 µm.
