# Supplementary material for: HIV-2-Infected Macrophages Produce and Accumulate Poorly Infectious Viral Particles
Source: Front Microbiol. 2020 Jul 10;11:1603. doi: 10.3389/fmicb.2020.01603 (PMC7365954; doi:10.3389/fmicb.2020.01603)
Supplement: Supplementary file 5 [file Image_5.pdf]

Supplementary Figure S5

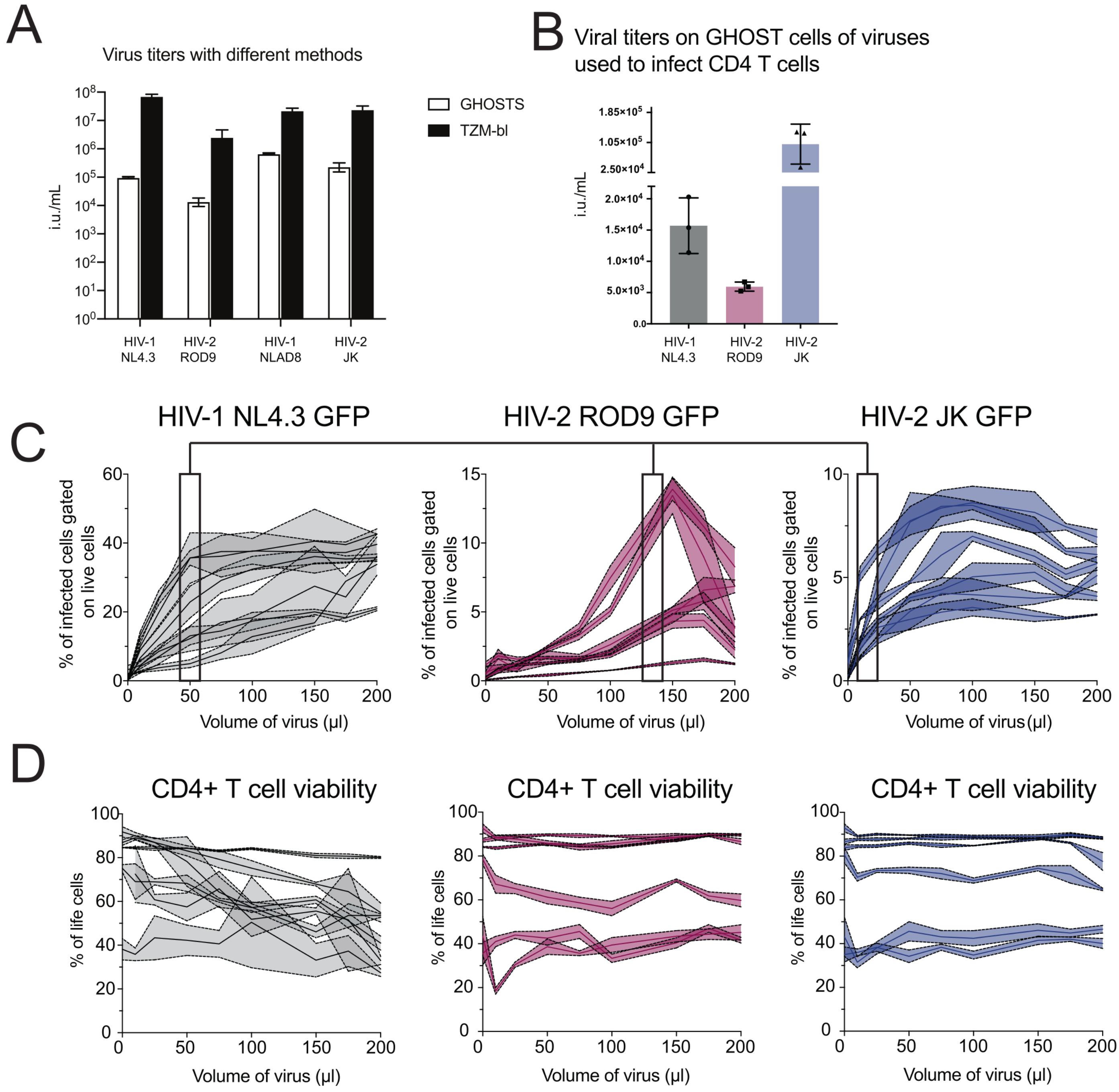

Figure S5. HIVs produced in HEK293FT titrated in reporter cells and primary CD4+ T cells. (A) The different HIVs used were titrated in both GHOST and TZM-bl reporter cell lines at the same time. Titres were calculated by the slope obtained by different volumes of virus on the reporter cells and the readouts for each reporter (GFP+ cells on FACS for GHOST cells and luminescence for TZM-bl cells). **(B, C and D)** CD4+ T cells were infected with non-pseudotyped HIVs produced by HEK293FT, n=6 donors and 3 independent experiments. **(B)** Viral titres on GHOST cells of the viruses used to infect human primary CD4+ T cells. The viruses were used fresh and titrated in parallel on GHOST and CD4+ T cells. **(C)** CD4+ T cell infections by different volumes of the indicated viruses. At 3dpi CD4+ T cells were recovered and analysed by flow cytometry. Similar rates of infections were obtained on GHOST cells for each of the 3 viruses using the volume of supernatant that are in the vertical linked boxes. **(D)** Cell viability of HIV exposed primary cells corresponding to experiments depicted in (C).
