# Supplementary material for: HIV-2-Infected Macrophages Produce and Accumulate Poorly Infectious Viral Particles
Source: Front Microbiol. 2020 Jul 10;11:1603. doi: 10.3389/fmicb.2020.01603 (PMC7365954; doi:10.3389/fmicb.2020.01603)
Supplement: Supplementary file 6 [file Image_6.pdf]

Supplementary Figure S6

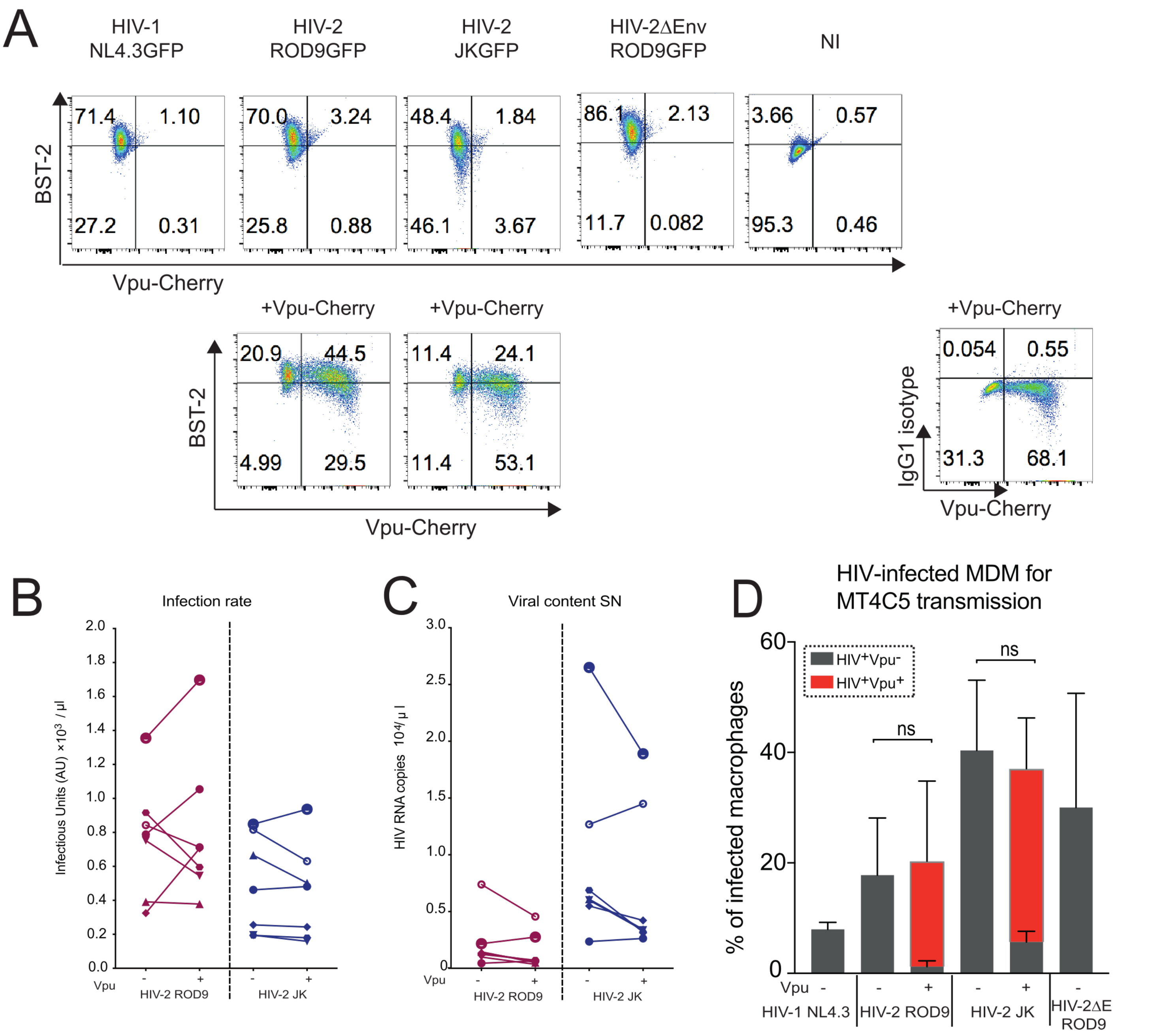

Figure S6. Expression of HIV-1 Vpu in HIV-2-infected MDMs does not impact the release of viral particles nor their infectivity. (Correspond to Fig. 7). MDMs were infected with the indicated virus or not (NI= non-infected), supplemented with lentiviral particles encoding the fusion protein Vpu-Cherry. At 3 dpi, cells were recovered and stained for BST2 and analysed by flow cytometry. **(A)** Representative dot plots for Vpu-Cherry and BST2 expression are presented. Data are representative of 7 donors from 4 independent experiments. Percentages of cells are indicated in the quadrants. **(B)** Infection rate of the particles released at 3 dpi in the supernatant of infected MDM +/- Vpu evaluated by titration on TZM-bl reporter cell line (see methods). **(C)** Release of viral RNA copies at 3 dpi in the supernatant of infected MDM +/- Vpu evaluated by RT-qPCR. **(D)** Rates of infection of MDMs corresponding to Fig 7D were evaluated by measuring the percentage of GFP+ MDM exposed or not to Vpu-Cherry lentivirus. Percentages of GFP+ cells and of GFP+Vpu+ (in red) are displayed.
